# Clinical Features, Treatment, and Outcomes of Mucormycosis: A Retrospective Analysis of 13 Cases from a Single Center in Turkey (2015–2025) †

**DOI:** 10.3390/tropicalmed11060152

**Published:** 2026-06-03

**Authors:** Esma Kepenek Kurt, Rukiyye Bulut, Bahar Kandemir, İbrahim Erayman

**Affiliations:** Department of Infectious Diseases and Clinical Microbiology, Faculty of Medicine, Necmettin Erbakan University, Konya 42090, Turkey; dr.rukiyye@hotmail.com (R.B.); tekinbahar@hotmail.com (B.K.); drerayman@yahoo.com (İ.E.)

**Keywords:** mucormycosis, predisposing factor, ROCM

## Abstract

Mucormycosis is a rare, invasive fungal infection associated with high mortality. This retrospective study evaluated adult mucormycosis cases followed in our clinic between 2015 and 2025. A total of 13 patients were included, with a mean age of 50.92 ± 20.65 years (range, 20–83), and 10 (76.9%) were male. The most frequent symptom was facial pain (100%). Four (30.8%) of the patients had rhino-sinusitis, and three (23%) had rhino-orbito-cerebral involvement. Seven (53.9%) of the patients had hematological malignancy, three (23%) had diabetes mellitus (DM), two (15.4%) had a history of COVID-19 pneumonia, and one (7.7%) had a history of both DM and COVID-19 pneumonia. All had elevated C-reactive protein levels. *Rhizopus* spp. grew in nasal/tissue cultures of three (23%) patients, and so did *Mucor* spp. Surgery was performed in 11 (84.6%) patients, and fungal hyphae were observed in tissue histopathology. All patients showed radiological findings of mucormycosis on imaging. All patients received Liposomal Amphotericin B, and nine (69.2%) patients received sequential posaconazole therapy. Recurrence occurred in two (15.4%) patients. A total of eight (61.5%) patients, including three (23%) patients with intracranial involvement, died. Mucormycosis is a severe infection, especially in patients with hematological malignancies or DM, despite early diagnosis and combined antifungal and surgical treatments.

## 1. Introduction

Mucormycosis is a rare but often fatal invasive fungal infection caused by fungi belonging to the order Mucorales. Infectious agents include species such as *Rhizopus* spp. and *Mucor* spp., which are commonly found in the environment, and these microorganisms can be found in soil, decaying organic material, and moist environments. The primary ways infection occurs are by breathing in Mucorales spores through the respiratory system, ingesting contaminated food, or through exposure via skin injuries. Because mucormycosis is a disease caused by Mucorales and other Zygomycota fungi, it is sometimes referred to as zygomycosis in the literature [1,2,3].

Mucormycosis is classified into several clinical forms, including pulmonary mucormycosis (PM), rhino-orbital-cerebral mucormycosis (ROCM), disseminated mucormycosis, cutaneous or soft tissue mucormycosis, gastrointestinal mucormycosis, and other less common types such as renal infection, endocarditis, osteomyelitis, and peritonitis [2].

ROCM creates a dramatic clinical picture characterized by progressive tissue invasion by infectious agents, starting from anatomic sites such as the nasal mucosa, paranasal sinuses, and palate. Agents can penetrate mucosal barriers, travel through surrounding tissue, nerves, blood vessels, and fascial planes, often reaching vital structures at the base of the brain. Mucormycosis agents are particularly notable for their ability to invade blood vessels in tissues such as the paranasal sinuses, lungs, and gastrointestinal tract. This can lead to thrombosis, necrosis, and mycotic thrombus formation in vascular walls, leading to hematogenous spread of infection. Particularly, diabetic ketoacidosis is strongly associated with the ROCM form of mucormycosis. Clinically, ROCM can present with atypical symptoms, such as nasal congestion, crusting, headache and facial pain, proptosis, chemosis, edema, ptosis, and ophthalmoplegia, resembling complicated sinusitis. Black necrotic lesions may be observed on the hard palate or in the nasal cavity, but this is not typical. The disease typically begins with a headache and can rapidly progress to a severe course, including orbital cellulitis, cranial nerve palsies, vascular thrombosis, coma, and death [4].

Although mucormycosis is rare, it can have a fatal course. The aim of this study is to retrospectively evaluate in detail the risk factors, clinical features, laboratory parameters, diagnostic methods, and treatment outcomes of adult mucormycosis cases followed in our clinic, and to contribute to the diagnosis and management of the disease in light of the findings obtained.

## 2. Materials and Methods

Approval was obtained from our university ethics committee for this study (decision no. 2025/5685). Informed consent was obtained from all patients or their relatives for the use of patient information and photographs. Patients followed in our clinic with a diagnosis of mucormycosis between 2015 and 2025 were then evaluated. The study’s starting year was determined as 2015 because reliable and complete access to our Hospital Information Management System records is only available retrospectively up to 2015. Adult patients (≥18 years) followed in our clinic with a confirmed clinical, radiological, and/or microbiological/histopathological diagnosis of mucormycosis were included. Patients with insufficient medical records and those with microorganisms that colonized in culture were excluded. Demographic characteristics of the patients included in the study (age, gender), site of disease involvement (rhino-cerebral, pulmonary, etc.), underlying predisposing risk factors [diabetes mellitus (DM) or hematological malignancy, etc.], laboratory parameters at diagnosis [erythrocyte sedimentation rate (ESR), C-reactive protein (CRP), procalcitonin (PCT), neutrophil and leukocyte count, etc.], imaging findings [computed tomography (CT) or magnetic resonance imaging (MRI)], whether surgical intervention was performed, diagnostic methods (culture, histopathological examination, etc.), drugs used in antifungal therapy and duration of treatment, and disease prognoses were obtained by reviewing the hospital information management system and patient files. Neutropenia was defined as a neutrophil count below 500 cells/mm^3^ at the time of diagnosis. The data obtained from the research were transferred to a computer environment and analyzed using the Statistical Package for Social Sciences (SPSS, IBM, Chicago, IL, USA) 22 software package. In data analysis, continuous variables were presented as mean ± standard deviation or median, and categorical variables were presented as number (n) and percentage (%). The normality of the data distributions was evaluated using the Shapiro–Wilk test and visual methods (histograms and probability plots). The Mann–Whitney U test was used to compare two independent groups where the data did not conform to a normal distribution. Fisher’s exact chi-square test was used to compare categorical data. The statistical significance level was accepted as *p* < 0.05.

## 3. Results

The mean age of the 13 patients included in the study was 50.92 ± 20.65 years, with an age range of 20–83. Ten (76.9%) of the patients were male. The most common symptom was facial pain (100%). The most common symptom associated with ocular involvement (84.6%) was periocular swelling. The patients’ clinical symptoms at admission are shown in Table 1. One case was diagnosed in 2015, one case in 2017, four cases in 2021, one case in 2023, three cases in 2024, and three cases in 2025.

**Figure 1:**
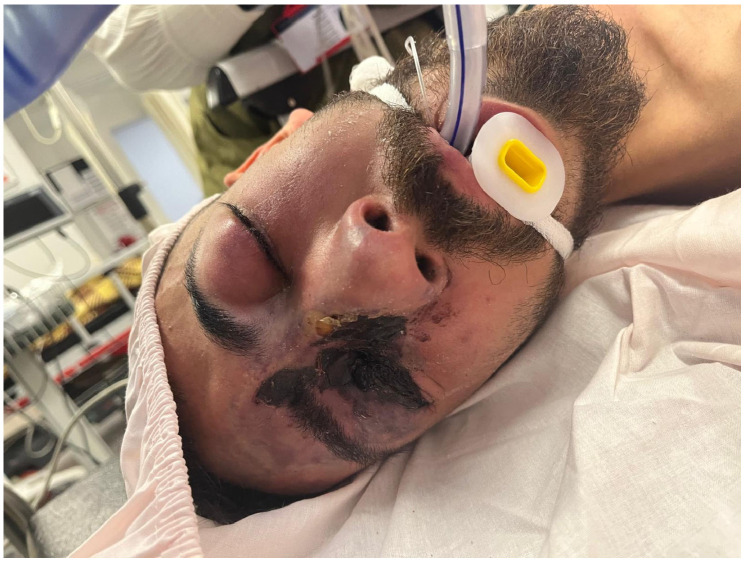
Necrotic plaque lesion around the eye with black eschar tissue on its surface. There are edema, erythema, and swelling in the left eye.

**Figure 2:**
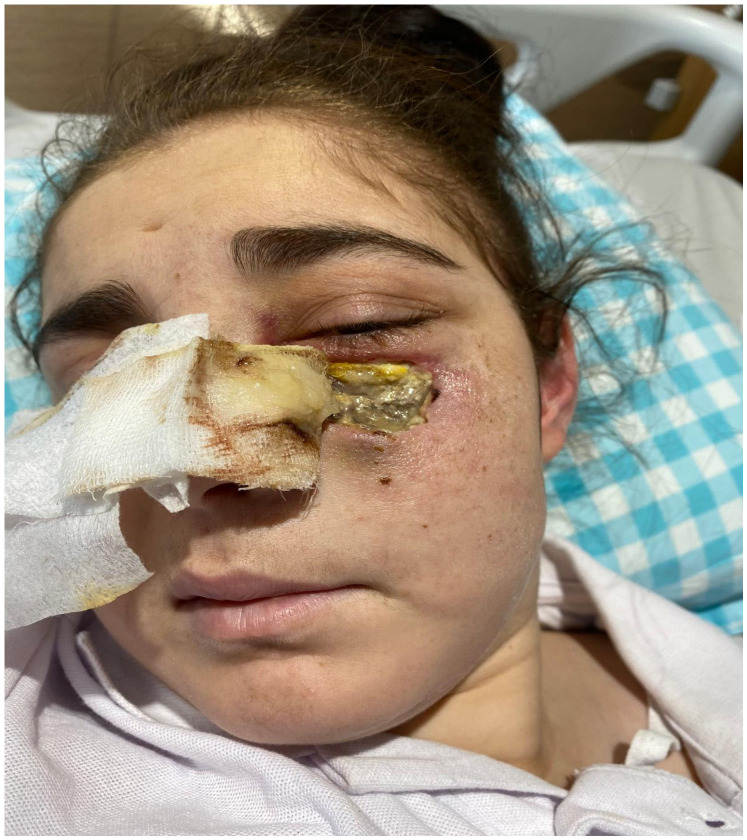
There is a broad, ulcerated, decayed purulent plaque with sharply defined borders in the left malar region. Redness and increased heat around the eyes (preseptal cellulitis).

**Figure 3:**
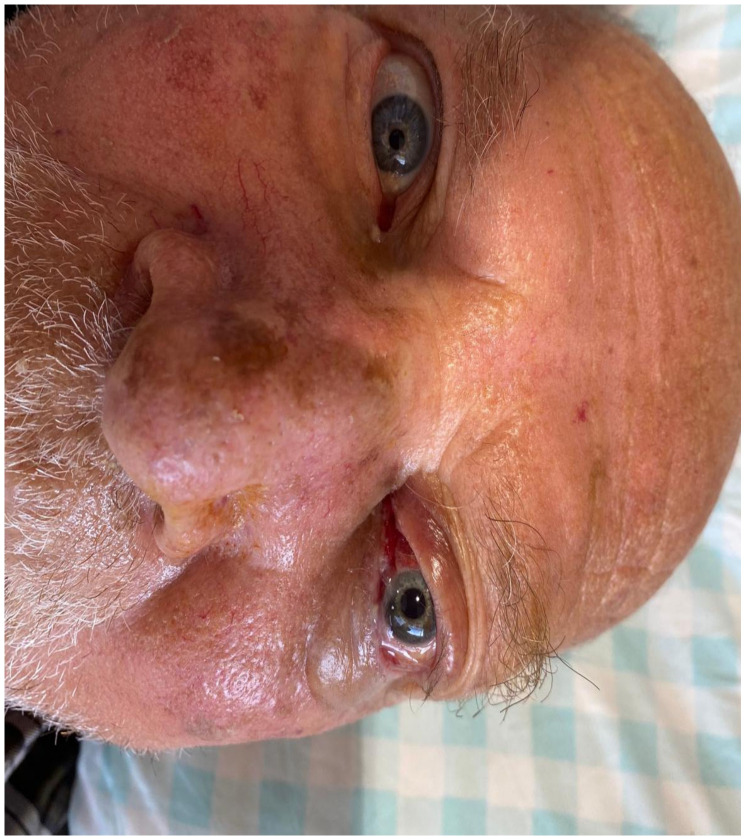
There is a thin, skin-colored plaque with patches of brown crust on the nose, hyperemia in the left eye, increased temperature, and edema on the left cheek and nose.

**Figure 4:**
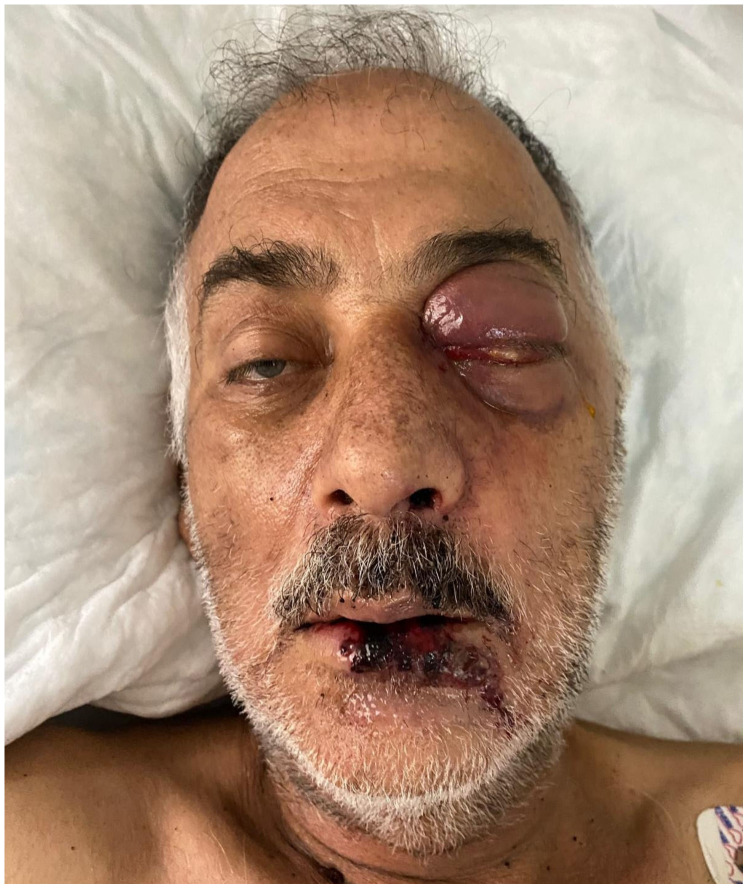
There is a black, necrotic plaque lesion around the mouth that spreads to the surrounding area. There are erythema, edema, and swelling in the left eyelid.

When the locations of involvement were examined, four (30.8%) patients had rhinosinusitis, three (23%) had ROCM involvement, two (15.4%) had rhinosinusitis-orbital involvement, two (15.4%) had rhino-orbital involvement, one (7.7%) had sinusoidal involvement, and one (7.7%) had mandible involvement. The first patient is shown in Figure 1, the second patient in Figure 2, the fourth patient in Figure 3, and the fifth patient in Figure 4. When predisposing factors were evaluated, seven (53.8%) patients had a hematological malignancy (two had a history of allogeneic bone marrow transplantation), three (23%) had DM, two (15.4%) had a history of coronavirus disease 2019 (COVID-19) pneumonia, and one (7.7%) had a history of both DM and COVID-19 pneumonia. The locations of involvement and predisposing factors are shown in Table 2.

At the time of diagnosis, the median (Q1–Q3) hemoglobin (Hg) values of the patients were 9.43 g/dL (7.1–12.55 g/dL), the median (Q1–Q3) leukocyte count was 6.45 × 10^3^/µL (0.23–14.9 × 10^3^/µL), the median (Q1–Q3) neutrophil count was 5.4 × 10^3^/µL (0.11–13.6 × 10^3^/µL), the median (Q1–Q3) lymphocyte count was 0.41 × 10^3^/µL (0.11–1.8 × 10^3^/µL), the median (Q1–Q3) platelet count was 161 × 10^3^/µL (42–252 × 10^3^/µL), median (Q1–Q3) ESR was 37 mm/h (n = 9, 19–75 mm/h), and the median CRP (Q1–Q3) was 134 mg/dL (83–217.5 mg/dL). Median (Q1–Q3) PCT was 0.52 µg/L (0.18–1.47 µg/L). At the time of diagnosis, anemia was detected in 10 (76.9%) patients, leukopenia in five (38.5%), neutropenia in five (38.5%), lymphopenia in eight (61.5%), thrombocytopenia in six (46.2%), leukocytosis in five (38.5%), and neutrophilia in five (38.5%) patients. ESR was measured in seven patients. Laboratory parameters at admission are shown in Table 3. The first patient was diagnosed with DM and had a glycosylated hemoglobin A1C (Hb A1C) level of 14.6. The fourth patient, also with DM, had an Hb A1C value of 11.4, the sixth patient had an Hb A1C value of 12.3, and the tenth patient had an Hb A1C value of 7.5. In patients who followed up for *Mucor* infection, the median age of surviving patients was found to be statistically significantly lower than the median age of deceased patients (*p* = 0.040). However, when comparing the laboratory parameters of surviving and deceased patients, no statistically significant difference was found between the groups (the Mann–Whitney U test was performed). Among patients followed up for *Mucor* infection, no statistically significant differences were found between surviving and deceased cases in terms of gender (*p* = 0.51), predisposing factors including history of COVID-19 pneumonia (*p* = 0.685), presence of neutropenia (*p* = 0.565), history of transplantation (*p* = 0.510), presence of hematological malignancy (*p* = 0.587), presence of DM (*p* = 0.49), and history of voriconazole prophylaxis use (*p* = 0.51). CT and MRI findings of the patients are shown in Table 4.

All patients received initial Liposomal Amphotericin B (L-AMB; 5–10 mg/kg/day), and the mean treatment duration was 54.7 ± 41.1 days (minimum–maximum 3–122 days). Posaconazole was administered as maintenance therapy to patients 1, 2, 4, 5, 6, 7, 11, 12, and 13. The total mean duration of posaconazole treatment was 61.8 ± 49.5 days (n = 8, minimum–maximum; 5–165 days). The second patient was treated for a bacterial infection caused by preseptal cellulitis. The third patient was diagnosed with mucormycosis based on clinical and laboratory findings, but died on the third day of L-AMB treatment before surgery could be performed. The sixth patient received L-AMB for 51 days and underwent endoscopic sinus surgery. He was discharged with oral posaconazole on the 18th day of posaconazole treatment. However, as he was a non-compliant patient, he failed to return for follow-up and presented with a relapse, and underwent left eye enucleation. Following the relapse, he received L-AMB for 36 days and posaconazole for 41 days. The ninth patient underwent surgery for a perianal abscess on the fifth day of mucormycosis diagnosis before debridement could be performed and died from a postoperative bloodstream infection. The thirteenth patient underwent endoscopic sinus surgery after receiving L-AMB for 56 days and posaconazole for 94 days due to relapse and brain involvement. Because surgery was not feasible for the brain lesions, caspofungin was added, and the patient died on the 59th day of L-AMB recurrence. A total of 11 (84.6%) patients underwent surgical intervention and debridement, including nine (69.2%) patients (excluding patients 1, 3, 7, and 9) who underwent endoscopic sinus surgery. Eight (61.5%) patients died.

## 4. Discussion

ROCM is the most common form of mucormycosis in humans [5]. Intra-abdominal, hepatic and gastrointestinal mucormycosis are less common than others, but they have a high mortality rate [4,6]. In multicenter studies, ROCM usually accounts for 60–70% of cases, pulmonary disease for 10–15%, and disseminated disease for 5–6% [7]. In our study, rhinosinusitis was the most common site of involvement in four (30.8%) patients, and one had pulmonary involvement. The lesion in the lung of the 12th patient, who had a cavitary nodular lesion in the right lung on thoracic CT, was initially suspected of being pneumonia caused by mucormycosis. Although radiologically consistent, histopathological sampling was not performed. The radiographic presentation of PM is broad, including focal consolidation with nonspecific infiltrates, cavitary lesions, or even diffuse opacities. In more immunocompetent hosts, PM may present with more atypical, slowly progressing forms. Asymptomatic solitary nodules have been described. Patients with DM have a predilection for developing endobronchial lesions that present with a less fulminant course than PM encountered in the neutropenic or transplant population [4].

In the study by Demiroglu et al., it was found that ROCM cases were more common in men (55.1%), and the average age was 56.6 ± 15.5 years [8]. In our study, we observed a predominance of male patients, and the patients who died were older than the patients who survived.

The most frequently observed findings in the studies were facial swelling, sagging, vision loss, nasal necrosis, and hard palate necrosis [8]. In our study, patients also exhibited different symptoms, primarily facial pain. Vision loss occurred in six patients, with three having rhino-orbital involvement and three having ROCM involvement.

Common risk factors for mucormycosis include DM, hematological malignancy, hematopoietic stem cell transplantation, solid organ transplantation, long-term neutropenia, corticosteroid use, and deferoxamine therapy [2]. In the study by Demiroglu et al., DM was identified as the most frequently detected underlying disease, at 65.3%, and Hb A1C values were determined to be high and DM was diagnosed in three out of 32 cases (9.3%) upon presentation to the ROSM clinic [8]. In our study, Hb A1C values were also found to be high in DM patients and only one patient (7.7%) was diagnosed upon presentation. This result indicates that the DM status in our patients is uncontrolled.

Hematologic malignancies are the second most common (33%) underlying cause of mucormycosis, with the disease being reported most frequently in patients with AML (Jeong, 2019 [7]). In our study, the most frequent risk factor for mucormycosis was hematological malignancy in 7 (53.8%) patients, and the most common hematological malignancy was ALL. Invasive pulmonary aspergillosis (IPA) is common in patients with hematologic malignancies. Prolonged neutropenia, chemotherapy, hematologic malignancies, hematopoietic stem cell transplantation, GCHD, solid organ transplantation, and prolonged high-dose corticosteroid therapy are also risk factors for IA. IA in patients with cirrhosis or liver disease is associated with very high mortality [9].

Following an increase in mucormycosis cases in early 2021 during the second wave of the COVID-19 pandemic, COVID-19 was identified as a new risk factor for mucormycosis [10]. In our study, most patients (84.6%) were followed up in the post-pandemic period. Hypoxia, high glucose levels, immunosuppressive diseases, immunosuppressive therapies, including corticosteroids, and prolonged hospitalization in individuals with COVID-19 facilitate the establishment and proliferation of Mucorales spores. Oxygen administered via the nasal canal can cause dryness and tissue damage in the nose and sinuses. This can increase the risk of mucormycosis by facilitating the growth of Mucorales fungi in a high-oxygen environment [11,12]. In our study, the second patient had a history of kidney transplantation six months prior, a recent intensive care unit admission for COVID-19 pneumonia 18 days prior to admission, and the use of corticosteroids and anakinra. The fourth patient had a history of DM, and hospitalization and corticosteroid treatment 20 days prior for COVID-19 pneumonia. The fifth patient had COVID-19 pneumonia 14 days prior to hospitalization. COVID-19 viruses can cause IPA [9].

Furthermore, the use of antifungal drugs such as fluconazole, voriconazole, and caspofungin, which are not effective against mucor, may be associated with the development of mucormycosis [2]. Voriconazole prophylaxis was administered to three of the patients with hematological malignancies before diagnosis.

In a study of 31 patients diagnosed with mucormycosis, the median leukocyte count at disease onset was 0.5 × 10^9^/L, the median neutrophil count was 0.09 × 10^9^/L, and the median platelet count was 22 × 10^9^/L. CRP was high in all patients. Although PCT levels were higher in patients who died than in patients who recovered, this difference was not statistically significant [13]. In our study, laboratory parameters in mucormycosis were nonspecific.

Microscopic examination of fungal hyphae is a simple diagnostic method for mucormycosis. Smears are prepared using potassium hydroxide (KOH) and calcofluor white. The hyphae’s morphology shows a ribbon-like structure, 5–25 μm in diameter, with irregular width and right-angled branches. Diagnosis of mucormycosis is difficult in fragmented hyphae; therefore, infection must be confirmed by a culture test. But positive cultures for the organisms that cause mucormycosis should be interpreted with caution, because these organisms are ubiquitous in the environment and can be detected in patients without the disease. Serological tests for diagnosis are not available. Histopathology and culture can be used for direct examination of tissue biopsies, sinus biopsies, and orbital tissue biopsies [4,7,14]. Hyphal structures were detected in the KOH stain of the nasal swab from the eighth patient. In our study, the diagnosis was made by culture growth in four cases. Culture positivity was particularly decisive for *Rhizopus* spp., but in some patients, despite no culture growth, the diagnosis was confirmed by histopathological confirmation. In our study, histopathological examination could be performed in 11 of the 13 patients, and fungal invasion was detected. Studies on mucormycosis report an association with *Aspergillus* [15]. In our study, *Aspergillus* spp. were observed in the wound culture of patient 12.

CT and MRI methods play a crucial role in the diagnosis of mucormycosis, defining the extent of involvement, which is crucial for effective debridement of infected tissue. CT scans provide better detection of bone erosion. When orbital and intracranial involvement and vascular structures are present, MRI visualizes them better [14,16]. In this study, when CT and MRI findings were evaluated in terms of mucosal thickening in the sinuses, orbital and brain involvement, and spread to areas such as the cavernous sinuses and meningeal structures, it was observed that the disease frequently begins and progresses in the orbit and paranasal sinuses. Particularly in cases with ROCM, MRI findings suggestive of serious complications such as meningoencephalitis, septic embolism, and pial enhancement have been detected.

Lumbar puncture plays a critical role in the diagnosis of central nervous system mucormycosis. Typical cerebrospinal fluid (CSF) abnormalities include high opening pressure, lymphocytic pleocytosis, high protein concentration, and low glucose levels [17]. In our study, CSF examination in patient 1 revealed a cloudy appearance, 187 cells/mm^3^, 95% neutrophils, CSF protein 171 mg/dL, glucose 92 mg/dL, and chloride 170 mmol/L, consistent with meningitis. Because the CSF samples from patients 5 and 13 were insufficient, cell counts and biochemical evaluations could not be performed. No growth was detected in the CSF of patients 1, 5, and 13, or in the blood cultures of all patients.

The facial manifestations of mucormycosis are often mistaken for soft tissue infection, leading to a delay in diagnosis [18,19]. The patients whose images were shared in our study demonstrate prominent skin involvement.

Standard treatment for mucormycosis includes the elimination of predisposing factors, surgical debridement, and antifungal therapy [14]. L-AmB is the first choice for an antifungal agent and is generally administered at high doses (5–10 mg/kg). After clinical improvement, treatment is generally transitioned to oral posaconazole and continued until clinical and radiological resolution, which may require several months [20,21]. In our study, all patients received L-AmB as initial treatment, and some continued oral posaconazole as maintenance therapy. Treatment duration varied according to individual response. The 13th patient relapsed during posaconazole maintenance, so L-AmB was restarted, and surgery was performed, and caspofungin was added as salvage therapy, but the patient died during treatment. Caspofungin is an alternative agent, and the combination of L-AmB and caspofungin can be given as salvage therapy in severe cases [22].

Despite optimal antifungal treatment, surgery is often necessary because vascular occlusion limits drug penetration into infected tissues. Surgical debridement should continue until viable tissue is reached and may need to be repeated. In severe ROCM cases, excision of nasal cartilage or the palate may also be required [14,23]. In our study, 11 patients underwent surgical debridement, and combined surgery plus antifungal therapy was associated with improved survival in two patients. Orbital exenteration may be required in cases with orbital progression [14].

The mortality rate of ROCM ranges from 25% to 62%. Prognosis is better in patients with disease confined to the sinuses, whereas orbital or cerebral involvement is associated with higher mortality [14,24]. Including the three poor-prognosis patients with intracranial involvement, a total of eight patients (61.5%) died. IPA studies in critically ill patients report a higher mortality rate (63–97%) than that observed in patients with hematological malignancies [9].

This study has several limitations. Its retrospective and single-center design and small sample size (n = 13) limit the generalizability of the findings. Only patients diagnosed and followed up in the infectious diseases clinic were included. The lack of molecular diagnostic confirmation (e.g., PCR or metagenomic next-generation sequencing) may have affected diagnostic accuracy. Furthermore, some patients lacked certain laboratory data, such as ESR. The absence of a standardized treatment protocol may have affected clinical outcomes.

## 5. Conclusions

Mucormycosis carries a high mortality rate (61.5%) despite L-AmB and surgery. Intracranial involvement and neutropenia were associated with poor outcomes. Early diagnosis, aggressive debridement, and correction of underlying immunosuppression remain critical.

## Figures and Tables

**Table 1:** Symptoms and clinical findings at admission.

	n (%)
Facial pain	13 (100)
Headache	12 (92.3)
Facial swelling	12 (92.3)
Facial redness	12 (92.3)
Necrosis in the nasal cavity	12 (92.3)
Swelling around the eyes	11 (84.6)
Runny nose	10 (76.9)
Proptosis	9 (69.2)
Ptosis	8 (61.5)
Fever	7 (53.8)
Ophthalmoplegia	7 (53.8)
Necrosis in the mouth or palate	6 (46.2)
Vision loss	6 (46.2)
Necrosis around the eyes	3 (23.1)

**Table 2:** *Mucor* involvement locations, predisposing factors, clinical features, and prognoses in patients.

Case No.	Involvement Site	Predisposing Factors	Culture Result	Surgical Intervention	Histopathology	Prognosis
1	Rhino-orbital-cerebral	Type 1 DM, Corneal transplantation, retinal surgery	Nasal culture: *Rhizopus* spp.	Left orbital exenteration, debridement	(+)	Exitus
2	Sinusoidal	Kidney transplantation, COVID-19 pneumonia	Wound culture: *Mucor* spp. growth	ESS, debridement	(+)	Cure
3	Rhino-sinusitis	AML, neutropenia	(−)	Not performed	(−)	Exitus
4	Rhino-orbital	Type 2 DM, COVID-19 pneumonia	(−)	ESS	(+)	Exitus
5	Rhino-sinusitis	COVID-19 pneumonia	Tissue culture: *Rhizopus* spp.	ESS	(+)	Exitus
6	Rhino-orbital	Type 2 DM	No growth	ESS, left eye enucleation	(+)	Cure
7	Mandible	ALL, neutropenia	No growth	Mandibular excision	(+)	Treatment continues
8	Rhino-sinusitis	MDS, ABMT	Nasal culture: *Rhizopus* spp.	ESS	(+)	Cure
9	Rhino-sinusitis	ALL, neutropenia	(−)	Not performed	(−)	Exitus *
10	Rhino-orbital-cerebral	Type 2 DM	(−)	ESS, Decompressive craniectomy	(+)	Exitus
11	Rhino-sinusitis-orbital	AML, ABMT, acute AGVHD	Tissue culture: *Mucor* spp.	ESS	(+)	Exitus
12	Rhino-sinusitis-orbital	ALL	Nasal culture: *Aspergillus* spp.	ESS	(+)	Cure
13	Rhino-orbital-cerebral	ALL	No growth	ESS	(+)	Exitus

DM: diabetes mellitus; ALL: acute lymphoblastic leukemia; ABMT: allogeneic bone marrow transplantation; MDS: myelodysplastic syndrome; AML: acute myeloid leukemia; AGVHD: acute graft versus host disease; ESS: endoscopic sinus surgery. * The cause of death was a bloodstream infection caused by *Pseudomonas aeruginosa*.

**Table 3:** Laboratory parameters of patients at the time of admission.

Case No.	Hg (g/dL)	Leukocytes (×10^3^/μL)	Neutrophils (×10^3^/μL)	Lymphocytes (×10^3^/μL)	Platelets (×10^3^/μL)	ESR (mm/h)	CRP (mg/dL)	PCT (µg/L)
1	14.3	29.92	25.79	3.8	264	37	334	2.8
2	11.3	6.45	5.99	4.3	225	32	125	0.2
3	5.6	0.2	0.1	0	3	99	240	3.6
4	15.3	22.34	19.58	0.66	181	21	83	0.3
5	13.2	13.4	12.6	0.37	161	48	54.1	0.3
6	11.9	16.4	14.6	1.1	288	51	249.6	0.1
7	8.1	0.15	0.02	0.11	45	-	142.2	1.7
8	7.8	9	5.4	2.51	240	-	175.5	0.1
9	9.4	0.1	0	0.11	48.3	8	87.3	1.2
10	9.8	4.4	2.9	0.88	352	126	24	0.17
11	6.6	0.27	0.13	0.08	18	-	195	0.66
12	6.8	10.5	8.8	0.41	139		83	0.71
13	7.4	0.57	0.16	0.32	39		134	0.52

Note: “-” indicates no data available. Hg, hemoglobin (12.1–17.2 g/dL); ESR, erythrocyte sedimentation rate (0–20 mm/h); CRP, C-reactive protein (0.1–5 mg/dL); PCT, procalcitonin (0–0.046 µg/L). Leukocytes (4–10 × 10^3^/uL), neutrophils (1.5–7.3 × 10^3^/μL), lymphocytes (0.8–5.5 × 10^3^/μL), platelets (150–400 × 10^3^/μL).

**Table 4:** Computed tomography and magnetic resonance imaging findings of the patients.

1 CT	Signs of fungal infection extending from the nasal cavity to the posterior ethmoid bones on the left.
1 MRI	Fungal infection extending from the left nasal region to the orbit and intracranial space (meningoencephalitis) and signs of embolic infarction.
2 CT	Soft tissue density is present in the extraconal area of the orbital floor on the left.
2 MRI	Signs of chronic sinusitis and preseptal cellulitis on the left.
3 CT	Retention cysts are seen in both maxillary sinuses and calcification in the falx.
3 MRI	Signs of chronic sinusitis and staphyloma are present in the left eye.
4 MRI	Signs of chronic sinusitis and inflammation extending to the orbit on the left.
5 CT	Inflammation and proptosis in the left preseptal and orbital areas.
5 MRI	Signs of inflammation extending to the orbit on the left.
6 CT	Signs of chronic pansinusitis on the left.
6 MRI	Signs of sinusitis and inflammation extending to the orbit on the left.
7 CT	Normal at diagnosis. During follow-up, osteonecrosis and pathological fractures were noted in the bilateral mandibular corpuscles and mandible.
7 MRI	Chronic osteomyelitis, necrosis, and inflammation in the adjacent soft tissues were observed on the right side of the mandible.
CT	Signs of pansinusitis and preseptal cellulitis on the right side.
8 MRI	Signs of sinusitis, preseptal, and orbital cellulitis on the right side.
9 MRI	Signs of fungal infection extending from the right nasolabial area to the nasal septum.
10 MRI	Right-sided pansinusitis, fungal infection findings extending to the orbital and intracranial areas (meningoencephalitis + cerebritis) and invading the right internal carotid artery, and embolic infarcts.
10 CT	The third ventricle is slightly enlarged. Calcification in the falx cerebri. Mucosal thickening in the right maxillary sinus.
11 CT	Signs of chronic pansinusitis and orbital cellulitis.
11 MRI	Fungal infection findings suggestive of pansinusitis and meningitis.
12 CT	Signs of left maxillary sinusitis, bony erosion of the left maxillary sinus wall, and fungal infection extending to the intratemporal fossa. Concurrent thoracic CT scan reveals cavitary nodules in the right lung.
12 MRI	Signs of left ethmoid and maxillary sinusitis, and fungal infection extending to the level of the pterygopalatine fossa, the buccal region, and the orbit.
13 CT	No bone destruction.
13 MRI	Findings of inflammation extending to the preseptal and retroorbital areas along with pansinusitis.

CT: computed tomography; MRI: magnetic resonance imaging.

## Data Availability

The original contributions presented in this study are included in the article material. Further inquiries can be directed to the corresponding author.

## Abbreviations

PM	pulmonary mucormycosis
ROCM	Rhino-Orbital-Cerebral Mucormycosis
DM	Diabetes Mellitus
Hg	Hemoglobin
ESR	Erythrocyte Sedimentation Rate
CRP	C-Reactive Protein
PCT	Procalcitonin
Hb A1C	glycosylated Hemoglobin A1C
CT	Computed Tomography
MRI	Magnetic Resonance Imaging
SPSS	Statistical Package for Social Sciences
COVID-19	Coronavirus disease 2019
ABMT	Allogeneic Bone Marrow Transplantation
MDS	Myelodysplastic Syndrome
AML	Acute Myeloid Leukemia
AGVHD	Acute Graft Versus Host Disease
ESS	Endoscopic Sinus Surgery
L-AMB	Liposomal Amphotericin B
IPA	Invasive pulmonary aspergillosis
KOH	potassium hydroxide
CSF	Cerebrospinal Fluid
PCR	Polymerase Chain Reaction
